# SOCRATES: Introducing Depth in Visual Wildlife Monitoring Using Stereo Vision

**DOI:** 10.3390/s22239082

**Published:** 2022-11-23

**Authors:** Timm Haucke, Hjalmar S. Kühl, Volker Steinhage

**Affiliations:** 1 Institute of Computer Science IV, University of Bonn, Friedrich-Hirzebruch-Allee 8, 53115 Bonn, Germany; 2 Senckenberg Museum for Natural History Görlitz, Senckenberg—Member of the Leibniz Association, Am Museum 1, 02826 Görlitz, Germany; 3 International Institute Zittau, Technische Universität Dresden, Markt 23, 02763 Zittau, Germany; 4 German Centre for Integrative Biodiversity Research (iDiv) Halle-Jena-Leipzig, Puschstrasse 4, 04103 Leipzig, Germany

**Keywords:** stereo vision, camera trapping, animal density, animal abundance, instance segmentation

## Abstract

The development and application of modern technology are an essential basis for the efficient monitoring of species in natural habitats to assess the change of ecosystems, species communities and populations, and in order to understand important drivers of change. For estimating wildlife abundance, camera trapping in combination with three-dimensional (3D) measurements of habitats is highly valuable. Additionally, 3D information improves the accuracy of wildlife detection using camera trapping. This study presents a novel approach to 3D camera trapping featuring highly optimized hardware and software. This approach employs stereo vision to infer the 3D information of natural habitats and is designated as StereO CameRA Trap for monitoring of biodivErSity (SOCRATES). A comprehensive evaluation of SOCRATES shows not only a 3.23% improvement in animal detection (bounding box ${\mathrm{mAP}}_{75}$), but also its superior applicability for estimating animal abundance using camera trap distance sampling. The software and documentation of SOCRATES is openly provided.

## 1. Introduction

The loss of biodiversity across a wide variety of ecosystems is accelerating. Ecologists need effective tools to monitor animal populations and address challenges such as climate and land use change, spreading of diseases, and invasive species. In recent years, camera traps have been instrumental in providing automated visual monitoring around the clock. However, all widespread camera traps are monocular, which prevents them from easily sensing their three-dimensional environment and the three-dimensional position and appearance of the observed animals.

As part of a German joint project on automated wildlife monitoring, Automated Multisensor stations for Monitoring of species Diversity (AMMOD) [1,2], we introduce SOCRATES to derive and use depth information (i.e., the distance between the camera trap and the observed scene) as a third dimension in addition to the regular two-dimensional image dimensions.

SOCRATES enables the following contributions:

- The detection and localization accuracy of animals is fostered by the additional depth information provided by SOCRATES (see Section 3.3 and Section 4.2).
- Abundance estimation traditionally uses methods such as camera trap distance sampling (CTDS) with commercial camera traps, which requires laborious manual workflows. SOCRATES instead provides depth information in a fully automated way using stereo vision (see Section 2.1 and Section 4).
- Reproducibility and accessibility for practitioners: The SOCRATES approach takes the practitioner’s perspective and provides our raw and labeled data (see Section 3.1), code, detailed instructions, best practices, and 3D CAD models.

## 2. Related Work

Related work is reported on from three perspectives. First, the principles of stereo vision and prior work on stereo camera traps are summarized. Second, recent progress with respect to visual object detection in images and video clips is reported. Third, an overview of approaches for estimating the density and abundance of unmarked animal populations using camera traps is given.

### 2.1. Depth Using Stereo Vision

Computer stereo vision is the well-established approach to image-based depth estimation. By comparing information about an observed scene from two differing camera perspectives, depth information can be derived. Usually, both cameras in a stereo setup are displaced horizontally from one another, yielding scene observations in terms of a left image and a right image. Computer stereo vision can be seen as the technical analogue to human stereopsis, that is human perception of depth and three-dimensional structure by combining visual information from two eyes. Depth is obtained from a stereo image pair by finding the distance, or disparity, between the corresponding projections of each scene point in the left and right images. Disparity *d* and depth *z* are inversely related, i.e.,

(1) $$z=\frac{b\;\cdot \;f}{d}$$

with *b* being the distance between both cameras (baseline) and *f* being the focal length of both cameras. To be able to efficiently find the projections of some scene point in both images, they first have to be rectified. Rectification results in projections of a single point to lie on a single horizontal scanline in both images and, therefore, reduces the dimensionality of the correspondence search from two to a single dimension. To obtain an accurate rectification, the intrinsic (internal camera parameters) and extrinsic (rotation and translation between the cameras) parameters have to first be obtained by a calibration procedure. For the calibration of the intrinsic parameters, a calibration object (e.g., checkerboard pattern printed on cardboard) has to be captured by the camera(s) to be able to associate 3D points in the scene with 2D points in the resulting image. To obtain the extrinsic parameters, eight or more correspondences between images of points in the projections of both cameras must be established [3]. Given a pair of rectified images, each of size W×H, stereo matching algorithms now estimate a cost volume *V* of size W×H×D. This cost volume contains for each pixel (i,j) and for each horizontal potential disparity between 0 and the maximum expected disparity D−1 some cost measure. The cost volume might be built by using traditional techniques such as the sum-squared difference between images a,b with patch height *c*, i.e.,

(2) $$V\left(i,j,d\right)=\sum_{\Delta j=-c}^{c}{\left(a\left(i,j+\Delta j\right)-b\left(i-d,j+\Delta j\right)\right)}^{2}$$

More recently, *V* has been estimated most successfully using learning-based approaches and convolutional neural networks [4,5,6,7,8]. We employed the *CREStereo* model [8] due to its demonstrated robustness in real-world conditions.

#### Stereo Camera Traps

A number of works have introduced camera traps with stereo camera setups [9,10,11]. The hardware of [9,10] is built around an FPGA, which controls two CMOS sensors, which are mounted vertically. A pyroelectric infrared sensor is connected to a microcontroller, which is, in turn, responsible for powering on the FPGA once motion is detected. The performance of the system is evaluated with respect to absolute size estimation of artificial and human targets. Reference [11] instead used an Intel RealSense D435 stereo camera, which computes stereo correspondence on the camera itself. However, due to the small baseline distance of roughly 5 cm, accuracy is limited at high distances. All prior works [9,10,11] were powered using mains electricity and were not optimized for being energy efficient and powered by a battery.

### 2.2. Instance Segmentation

State-of-the-art approaches to object detection are usually learning-based [12]. Given a 2D color or grayscale image, these methods learn to predict a set of bounding boxes, e.g., given by the 2D location of the upper-left and bottom-right corner of an axis-aligned rectangle fully enclosing the object [13]. Object detection methods might be extended to perform instance segmentation, where not only bounding boxes are predicted, but whether any pixel in the image belongs to the respective object (binary masks) [14]. Deep learning object detection and instance segmentation models usually consist of two parts. The backbone takes in the original image and produces a hierarchy of feature maps that encode higher-level information about the image. These feature maps are then used by the object detection or instance segmentation model to predict the bounding boxes and binary masks themselves [15]. A general issue of deep learning models is that their training requires immense amounts of annotated training data. Annotated data are raw data associated with corresponding labels, which can be of different modalities (e.g., object classes occurring in the image, bounding boxes around objects of interest, pixelwise masks of such objects, etc.). These labels must often be created manually and are therefore costly to obtain. This requirement of large annotated training datasets is slightly relaxed by transfer learning [16]. In transfer learning, the backbone is first pre-trained to perform some task involving a very large training dataset, e.g., performing image classification on ImageNet [17]. Visual concepts learned by such backbones have been shown to be generally useful and not just applicable to the pre-training task [18]. The backbone is then fine-tuned on the target task, which usually involves a much smaller dataset. Backbones are usually only pre-trained on 2D RGB images, as these are ubiquitous. However, recently, *Omnivore* [19] was introduced. Omnivore is a method to train a shifted window transformer [20] backbone on any combination of congruent color and depth images. We therefore used Omnivore to serve as a depth-aware backbone in our instance segmentation model.

### 2.3. Abundance Estimation

There exist a number of methods to estimate the density and abundance of unmarked animal populations using camera traps: the random encounter model (REM) [21], the random encounter and staying time model (REST) [22], the time-to-event model (TTE), the space-to-event model (STE), the instantaneous estimator (IS) [23], and camera trap distance sampling [24]. All of these require an estimation of the effective area surveyed by the camera trap. This area is not simply given by the optical constraints of the camera; instead, it is influenced by factors such as environmental occlusion and the range of the passive infrared sensor, which may not perform consistently at all locations within the camera’s viewshed. The effective area surveyed is statistically inferred by using the distances of the observed animals. Although there are approaches that estimate these distances (semi) automatically [25,26], they either require the laborious capture of reference material [25] or might not generalize to extreme scenarios, such as very close-up scenes within 3 m of the camera [26,27].

## 3. Materials and Methods

**Figure 1:**
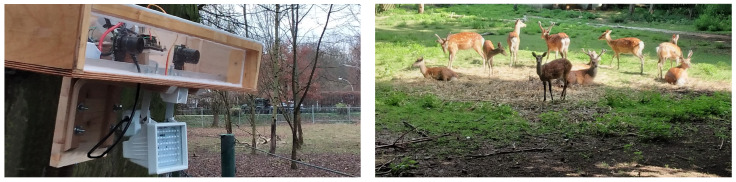
Introducing the SOCRATES camera trap system located in the wildlife park Plittersdorf located in Bonn, Germany, showing European fallow deer (Dama dama) and Sika deer (Cervus nippon).

The SOCRATES camera trap system comprises a cost and power efficient stereovision sensor platform, as well as state-of-the-art animal detection software based on a deep learning software architecture. The experimental evaluation of SOCRATES will utilize a representative dataset generated by SOCRATES in the wildlife park Plittersdorf, located in Bonn, Germany, exhibiting European fallow deer (Dama dama) and Sika deer (Cervus nippon). Figure 1 illustrates the deployment of SOCRATES in the wildlife park Plittersdorf.

### 3.1. Data Material

**Figure 2:**
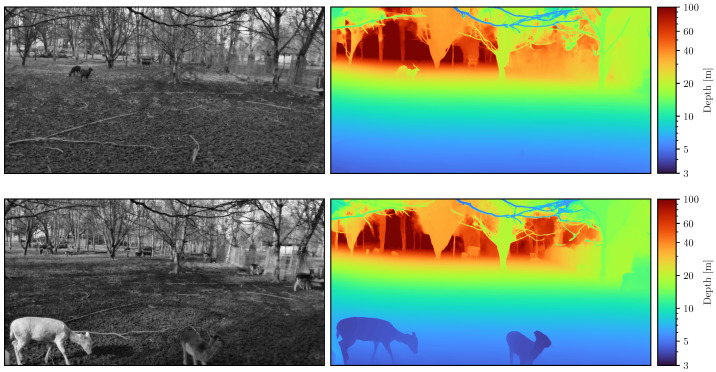
Samples of data collected in the wildlife park Plittersdorf. The left shows the grayscale image of the left camera, the right image the color-coded depth map obtained using stereo correspondence.

We deployed SOCRATES in the wildlife park Plittersdorf in order to evaluate the hardware and software. Details about this deployment may be found in Section 4. During this time, SOCRATES made 221 true positive observations. Exemplary samples are visualized in Figure 2. Each observation results in an HEVC encoded video with 30 frames per second and a length of 25 s. For our experiments, we sampled two sets of still images from these videos.

For our camera trap distance sampling study (see Section 3.4), we sampled still images from the videos at a rate of $2\;s^{-1}$, resulting in a total of 2871 images.

**Figure 3:**
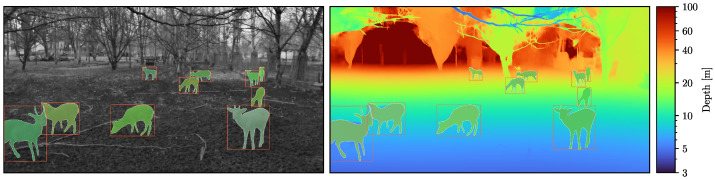
Examples of instance segmentation and bounding box annotations overlaid on top of the grayscale image of the left camera (**left**) and the color-coded depth map (**right**).

For the instance segmentation task (see Section 3.3), we wanted our dataset to consist of diverse scene configurations (animal positions and poses, lighting conditions, etc.). To obtain such diverse samples, sampling at regular intervals is not enough. Sometimes, deer will stand still for long periods of time, while moving quickly through the scene at other times. Therefore, we employed an approach based on background modeling using Gaussian mixture models [28]. We then accumulated the ratio of foreground pixels (that is, the ratio of pixels occupied by moving objects) in each video frame until a threshold of 10% was reached. This way, we sampled more often if there was more movement in the video and less often for less movement. We then annotated a total of 546 instances in 187 of the still images sampled this way with instance masks using the interactive annotation tool proposed by [29]. Figure 3 visualizes one of these annotated images. On average, we needed roughly 3.5 min per instance, resulting in a total annotation effort of roughly 32 h. Out of the total of 546, we used 395 instances for training and validation (via 10-fold cross-validation) and reserved 151 instances as the test dataset, such that images from a single video were only ever contained in one dataset. The test dataset was not used in this work, but was instead reserved for future work. We published both the raw data [30] and the instance segmentation dataset [31].

### 3.2. The SOCRATES Stereovision Sensor Platform

The SOCRATES stereovision platform is optimized for:

1. Operability;
  (a) At day and night time as well as;
  (b) For a wide range of animal-camera distances;
2. Effective and efficient power supply;
3. Hardware and construction costs;
4. Weather resistance.

**Figure 4:**
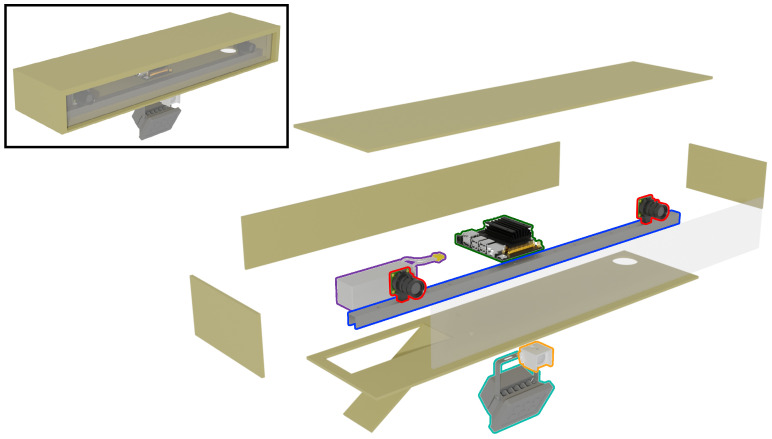
The 3D visualization of SOCRATES. The important components will be covered in the following text and are highlighted here, i.e., cameras (red outline), baseline rail (blue outline), control unit (green outline), battery (violet outline), infrared illumination (turquoise outline), passive IR sensor (Orange outline) Details such as the power supply, wiring, and screws are omitted. The following 3D parts of the model were obtained from external sources: Raspberry Pi HQ cameras [32], Jetson Nano Devkit [33], Infrared illuminator [34], PIR sensor case [35], LiPo battery [36].

Figure 4 illustrates the final hardware design, while the following section covers in detail the technical implementation and how we addressed these design goals. We first describe the stereo camera design (cameras and baseline, Design Goals 1 and 3). The raw data produced by the cameras were processed and stored by the control unit (Design Goals 2 and 3). Weather resistance (Design Goal 4) was provided by the case. Infrared motion detection and illumination facilitate energy efficiency (Design Goal 2) and operability at night time (Design Goal 1 (a)). We additionally describe in detail the power supply, how we obtained animal–camera distances using stereo correspondence, and how the captured data may be transferred using different connectivity options.

Cameras and baseline: A pair of Raspberry Pi high-quality cameras (Raspberry Pi Foundation, Cambridge, United Kingdom) were chosen for their cost-effectiveness and the high sensitivity of their Sony IMX477 sensor [37]. Interchangeable lenses allow adaptation to specific scenarios (i.e., shorter focal lengths for close-up scenes, higher focal lengths for more distant objects). Removal of the infrared filter allows sufficient exposure at night using artificial infrared illumination, while sacrificing color sensitivity. The cameras were mounted on a 77.5 cm-long U-shaped aluminum rail, which allowed the configuration of different baseline distances between both cameras.

Control: An NVIDIA Jetson Nano Developer Kit (NVIDIA Corporation, Santa Clara, United States of America) was used as the central control and storage unit. It is responsible for taking motion detection signals from the PIR sensor, turning on the power to the IR illuminator, and capturing, encoding, and archiving image material from the cameras. The raw RGB video material is encoded on the Jetson Nano’s GPU by synchronizing the left and right image streams and encoding the resulting video using the HEVC video codec [38].

Motion detection: Like most camera traps, SOCRATES utilizes a pyroelectric infrared (PIR) sensor (HC-SR501, Sertronics GmbH, Berlin, Germany) for detecting motion and, thereby, triggering capture.

Illumination: A 12 W, 850 nm infrared illuminator was employed to ensure properly exposed images at night without disturbing most species.

Power supply: All components were powered by a lithium ion polymer battery with a theoretical capacity of 236.8 Wh (16,000 mAh at 14.8 V).

Connectivity: SOCRATES may transmit the recorded data to local *AMMOD base stations* [1] via wireless LAN. The AMMOD base stations are able to cache the data and forward them to the web portal of the AMMOD project (see Section 4.5) once conditions are favorable (e.g., abundant energy is available via energy harvesting [2]). If no AMMOD base station is available, data can alternatively be transmitted directly to the AMMOD web portal via a cellular modem.

Stereo correspondence: The central goal of SOCRATES is to infer depth information through stereo vision. In the natural world, as well as in computer vision, this is achieved by solving the stereo correspondence problem (see Section 2.1). We performed both intrinsic and extrinsic calibration using *Kalibr* [39] with a grid of 4×3 AprilTags [40] mounted on a wooden board as the calibration target. During the setup of SOCRATES, the calibration target was manually moved through the scene such that it covered as much of each camera’s field of view as possible. After SOCRATES is assembled and calibrated, calibration does not have to be repeated when deployed to different locations, as the calibration is not dependent on a specific location, but only on the camera configuration. Given the intrinsic and extrinsic parameters, the images of both cameras are rectified, and the disparity of each pixel is computed using [8]. The rectification and disparity computation are performed on a separate GPU server to prolong the battery life of SOCRATES.

### 3.3. Depth-Aware Instance Segmentation

**Figure 5:**
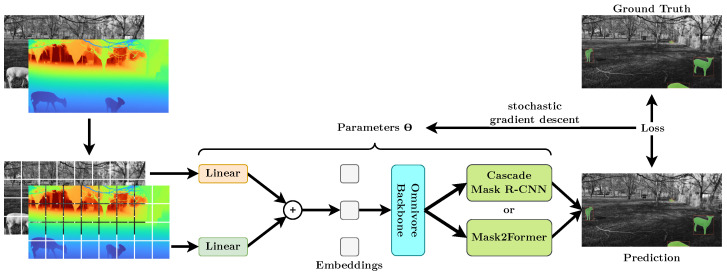
The data flow of our RGB-D instance segmentation model using Omnivore [19] and Cascade Mask R-CNN [42] or Mask2Former [15]. In Omnivore, the grayscale and depth images are first split into 2D patches, linearly embedded and added together. The resulting embeddings are then passed through the Omnivore backbone, which generates hierarchical feature maps. These feature maps are then used by Cascade Mask R-CNN or Mask2Former to perform instance segmentation. The entire model is optimized via stochastic gradient descent via the AdamW optimizer [47]. The model architectures and loss computation are described in detail in [15,42].

We frame the problem of detecting and localizing animals as an instance segmentation problem, with the goal of generating a bounding box and a binary mask for each animal instance. Compared to animal presence–absence classification, this approach allows both counting the exact number of animals present, as well as inferring the distance between animal and camera by applying the binary mask to the depth images obtained using stereo vision. However, the depth images themselves obtain useful information for differentiating multiple individual animals from themselves and the background. Still, it is not obvious how to use the depth images obtained from SOCRATES in this framework. Compared to datasets such as ShapeNet [41], we only have information from (effectively) a single perspective. We therefore argue that it is wise to treat the depth information as an additional channel in the two-dimensional image instead of working on point clouds or voxel grids, which increase the computational and memory requirements while largely foregoing the significant improvements being made in the area of 2D instance segmentation. Although most backbones are pre-trained on color images without depth information, a recent work proposed *Omnivore*, a vision transformer backbone trained on color and depth information [19]. In our experiments, we used *Omnivore* as our backbone of choice. As instance segmentation models, we used either the convolution-based *Cascade Mask R-CNN* [42] or the transformer-based *Mask2Former* [15]. This is motivated by the observation that vision transformers require more training data to perform well, compared to CNNs [43,44]. Therefore, Mask2Former performs very poorly when trained on small datasets such as the *Plittersdorf instance segmentation dataset*, while outperforming Cascade Mask R-CNN on larger datasets such as Cityscapes [45]. The resulting model architecture is visualized by Figure 5. To demonstrate that depth information is not only beneficial on the Plittersdorf instance segmentation task, we also evaluated improvements on the Cityscapes instance segmentation dataset. This is because the Cityscapes dataset is one of the only datasets that provides both depth information through stereo vision and a large amount of instance segmentation annotations. We implemented our instance segmentation pipeline using *mmdetection* [46] and largely kept the default hyperparameters of the mmdetection model implementations, including basic color, horizontal flip, and random cropping augmentations. We used the AdamW optimizer [47] with a global learning rate of $5N10^{-5}$ with a batch size of *N* and a weight decay of 0.05. We set N=2 for Mask2Former and N=6 for Cascade Mask R-CNN due to memory constraints. To conserve the battery life of SOCRATES, we ran the instance segmentation model on a dedicated GPU-equipped server (see Section 4.5).

### 3.4. Camera Trap Distance Sampling Study

It is now possible to combine the instance masks generated by the animal detection model (see Section 3.3) with the depth images obtained by stereo vision (see Section 2.1) to obtain the distances required for the camera trap distance sampling (CTDS) [24] abundance estimation method. To be able to use all observations without leaking information from the training dataset of our instance segmentation model, in this study, we used not the instance masks, but the bounding boxes of MegaDetector [48] and the sampling approach by [25]. To show the viability of this approach, we performed an exemplary estimation of the detection probability. We used 7 equally spaced distance intervals from 3 m to 11 m. As SOCRATES is mounted on a tree just outside the enclosure and at a height of 1.9 m, 3 m is the minimal distance where deer are certain to be visible. We did not re-scale the minimum distance, as deer might be present closer to the camera, but outside the field of view. We used the *Distance for Windows* software (version 7.4 [49]) and modeled the detection function using a uniform key function with a single cosine adjustment term.

## 4. Evaluation

We operated SOCRATES in the wildlife park Plittersdorf, Bonn, Germany, from February 9th to July 8th 2022, or 149 days. The wildlife park Plittersdorf houses exclusively European fallow deer (Dama dama) and Sika deer (Cervus nippon). The camera was mounted on the side of a tree using a lashing strap and not moved during the entire duration. During this time, SOCRATES experienced temperatures from $-4\;{}^{\circ }C$ to $38\;{}^{\circ }C$ and storms with wind speeds of $87\;km\;h^{-1}$ without issues. SOCRATES was without power or the software disabled due to maintenance for 46 days, resulting in a total number of 103 observation days. During this time, SOCRATES recorded 1089 observations. Out of these, 221 showed visible animals. This indicates a false positive rate of roughly 80%, which is in line with prior work concerned with commercial camera traps [50]. False triggers are primarily induced by (1) animals in the field of view of the PIR sensor, but outside of the fields of view of the cameras, (2) excessive infrared illumination by the Sun during daytime, or (3) by artificial light sources such as flood lights on nearby buildings. We manually removed all false positive observations from our dataset. Although this could easily be automated, e.g., by using the MegaDetector [48], we wanted to ensure that there were no persons in the final dataset and, therefore, screened the entire dataset manually.

We compare SOCRATES with the widely used commercially produced Reconyx HP2XC in Table 1. SOCRATES is significantly larger to support large baselines, while having significantly shorter battery life and slightly higher component costs. The infrared illuminator of SOCRATES operates at a slightly shorter wavelength, which might be visible for some animals. The infrared illuminator should therefore be replaced with a longer-wavelength version in the future. At the same time, SOCRATES not only provides depth information through stereo vision, but also allows recording video at high resolutions and frame rates for long durations, only limited by available storage space. The interchangeable lenses, as well as the configurable baseline construction allow for adaptation to specific scenarios, e.g., free fields, feeding places, animal crosses, green bridges, etc., where animals are observed at different distances.

We demonstrate that the stereo capabilities SOCRATES facilitate improved visual animal detection (see Section 4.2) and accurate abundance estimation using camera trap distance sampling (see Section 4.4). Depth information is also essential for obtaining absolute animal sizes (referenced as photogrammetry in ecology [52]), which is traditionally performed using laser rangefinders. Furthermore, depth information has been shown to improve the accuracy of animal tracking over 2D-only approaches [53,54]. SOCRATES cannot compete with commercially available camera traps in cost or battery life, but this was not our goal. Apart from the methodological improvements described above, SOCRATES fulfills three high-level goals:

1. It demonstrates that stereo camera traps are viable and worthwhile. We hope to convince commercial camera trap manufacturers to support stereo camera setups using off-the-shelf hardware.
2. It facilitates the verification of monocular approaches. For example, abundance estimation using camera trap distance sampling might be performed twice, once using monocular approaches [25,26] and once using SOCRATES. Both raw animal distances and the resulting animal densities might then be compared.
3. It allows generating training data for monocular depth estimation methods such as [55,56,57]. These approaches have been largely focused on human-centric scenes such as indoor and street scenes with a relatively simple geometry, which are highly unlike natural scenes such as forests. Gathering training data from natural scenes might help these methods generalize better to such scenes and, thus, allow monocular camera traps to more accurately estimate depth information in the future.

### 4.1. Stereo Correspondence

We evaluated the accuracy of SOCRATES quantitatively by comparing the depth obtained from stereo correspondence (see Section 2.1) with n=6 pointwise ground truth measurements *Y* obtained using a laser rangefinder. For the ground truth measurements, we picked distinct objects such as tree trunks to be able to accurately re-identify them in the SOCRATES images. As metrics, we employed the root-mean-squared (RMSE) and end-to-end point (EPE) errors in disparity space. We obtained a root-mean-squared error of 1.63 px and an EPE of 1.43 px, which are in line with prior work on comparable scenes [5,6].

We additionally evaluated the temporal stability of the depth maps using the temporal quality metric proposed in [58], which is defined as:

(3) $$E_{t}=\frac{1}{\left(N_{T}-1\right)N_{P}}\sum_{n=2}^{N_{T}}\sum_{\left(x,y\right)}\left|D\left(x,y,n\right)-D\left(x-m_{x},y-m_{y},n-1\right)\right|$$

where $N_{T}$ is equal to the number of frames in the input video, $N_{P}$ is the number of pixels in a single frame, D(x,y,n) is the scalar disparity at some pixel (x,y) at time *n*, and $m_{x},m_{y}$ is the optical flow from frame *n* to frame n−1, calculated using [59]. Using [8], we obtained $E_{t}=0.4439$, which is on par with the temporal error of the ground truth disparity in [58].

Figure 2 shows some exemplary pairs of near-infrared images and corresponding depth maps inferred by [8]. As can be seen, the depth maps generally represent the scene well and clearly highlight the boundaries of the deer.

**Figure 6:**
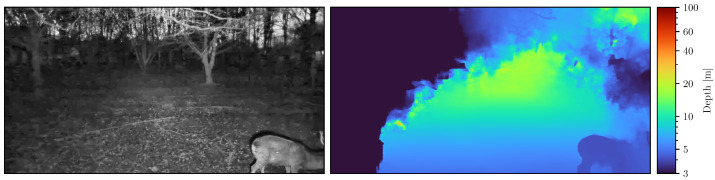
Stereo matching inevitably fails in regions where there is not enough available information.

Like regular camera traps, at night time, some regions in the field of view might be insufficiently lit and, therefore, underexposed in the resulting images. In these regions, insufficient image information is available to perform successful stereo correspondence, which is illustrated by Figure 6.

### 4.2. Visual Animal Detection

We used the COCO [60] metrics to evaluate our instance segmentation models. Each metric was obtained by performing 10-fold cross-validation after the last training epoch. Cross-validation is especially important in this setting, as it reduces the impact of a single lucky train–test split on this small dataset. Table 2 summarizes the results on the Plittersdorf instance segmentation task. The summarizing metrics for bounding boxes (${\mathrm{AP}}^{\mathrm{bbox}}$) and segmentation (${\mathrm{AP}}^{\mathrm{segm}}$) show that incorporating depth information results in an overall performance improvement. Interestingly, for low IOU thresholds (${\mathrm{AP}}_{50}^{\mathrm{bbox}}$, ${\mathrm{AP}}_{50}^{\mathrm{segm}}$), depth information seems to have the opposite effect. In other words, pure grayscale images perform better for roughly localizing an animal, whereas grayscale and depth information together are better for localizing animals very accurately ${\mathrm{AP}}_{75}^{\mathrm{bbox}}$, ${\mathrm{AP}}_{75}^{\mathrm{segm}}$). This is especially interesting as the ground truth labeling was performed using exclusively the grayscale image. Intuitively, one could therefore argue that the grayscale information is most important for matching the ground truth very precisely. Here, we see the opposite effect. As the error of stereo correspondence is quadratically related to the true distance, the resulting depth maps become less useful at larger distances. This is reflected in the lower performance on small instances (${\mathrm{AP}}_{s}^{\mathrm{segm}}$), which are typically farther away than medium (${\mathrm{AP}}_{m}^{\mathrm{segm}}$) or large instances (${\mathrm{AP}}_{l}^{\mathrm{segm}}$). We tried to ease the dependence on depth information for these faraway instances by clipping the depth values to different maximum distances or randomly dropping the depth information altogether during training [61]. However, this did not result in meaningful improvements. We provide benchmarks of inference and training time in Table A2 and an experimental comparison with more lightweight ResNet backbones in Table A1.

### 4.3. Depth-Aware Instance Segmentation on Cityscapes

To show that the positive effect of depth information on instance segmentation accuracy is not limited to settings with grayscale images, a single object class, and a fixed camera such as SOCRATES, we additionally evaluate our instance segmentation approach on the Cityscapes instance segmentation task [45]. The Cityscapes instance segmentation dataset [45] is composed of color and stereo depth images of urban street scenes, captured by cameras in a moving car. It features several object classes, such as *person*, *car*, or *bus*, annotated with instance labels. The Cityscapes dataset is also much larger, with 3475 annotated images in its training and validation sets. As can be seen in Table 3, the depth information has an overall even greater positive impact than in the Plittersdorf task (see Section 4.2). This is likely caused by two reasons: (1) the Mask2Former [15] being able to better make use of the depth-aware feature hierarchies produced by Omnivore [19] and (2) the larger training dataset, which might help alleviate the lower number of depth images during pre-training [19].

### 4.4. Abundance Estimation Using SOCRATES

**Figure 7:**
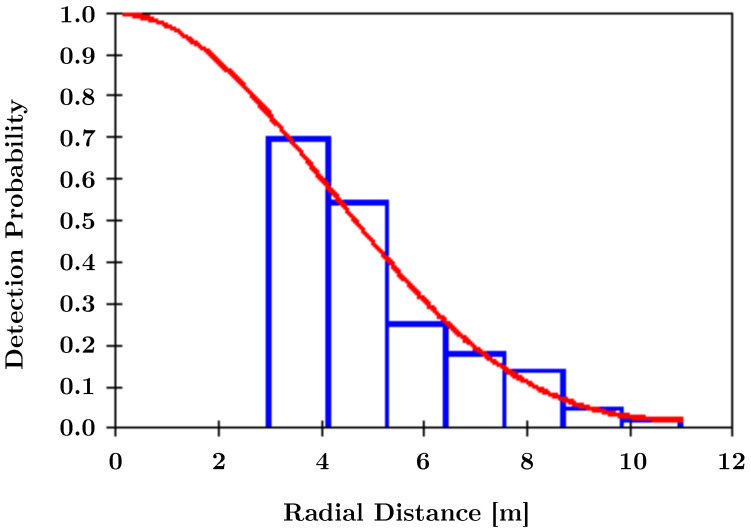
CTDS detection probability. We transformed our distance measurements into seven intervals (visualized in blue) from which the detection probability (visualized in red) was derived using CTDS.

Figure 7 depicts the detection probability obtained by CTDS using the parameters specified in Section 3.4. Note that the estimated probability density approximates the measurements well, starting from a distance of 3 m. Due to the way SOCRATES is mounted, deer below 3 m may not be visible, which is why we excluded these low distances from our estimation (see Section 3.4).

**Figure 8:**
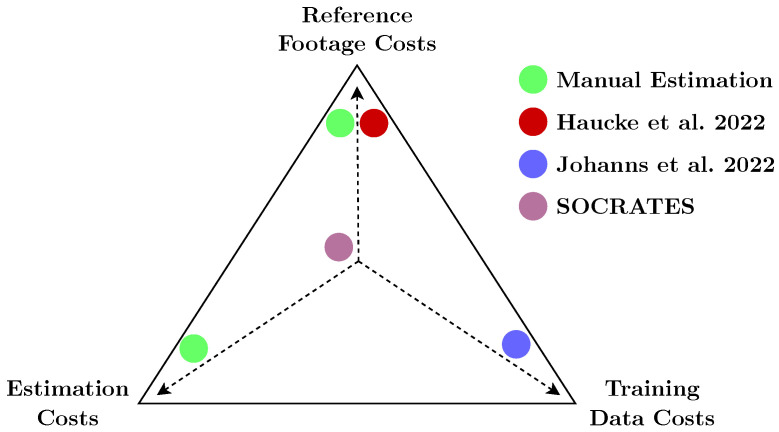
Tradeoff between reference footage costs, manual distance estimation costs, and training data costs. The manual approach requires both the acquisition of reference footage and labor-intensive manual animal distance estimation. Reference [25] requires reference footage, but the distance estimation itself is automated. Reference [26] requires neither reference footage nor manual distance estimations, but requires training data of similar scenes. SOCRATES measures distance using stereo vision and, therefore, incurs none of these costs.

Compared to competing approaches [25,26], distance estimation for abundance estimation of unmarked animal populations is straightforward with SOCRATES. Figure 8 visualizes the respective tradeoffs. The presented proof-of-concept for modeling detection probability in camera trap distance sampling with SOCRATES demonstrates the superiority of using SOCRATES in CTDS by significantly improving the efficacy of future wildlife surveys [25]. The reduced cost for data processing, the increase in animal detection, and potential for application in integrated mono- and stereo-camera trap surveys pave the way for an end-to-end solution in computational wildlife monitoring. The proposed approach is not limited to the conditions of our study, but is widely applicable across habitats, species, and regions. For future field surveys, we recommend that multiple SOCRATES devices be used, along with a random or systematic study design to estimate wildlife density and associated variance reliably. SOCRATES can also be paired cooperatively with traditional, monocular camera traps for improved error quantification and improvement of monocular distance estimations like that proposed by [26].

### 4.5. AMMOD Portal Case Study

**Figure 9:**
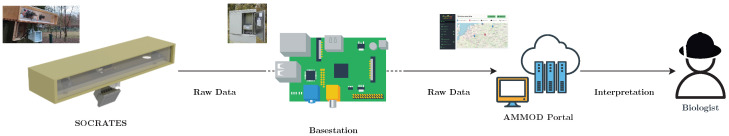
Fully automatic flow of data from SOCRATES over the base station to the AMMOD Portal and the expert end users. A GPU server runs the instance segmentation and distance estimation steps and uploads the results back to the AMMOD portal.

A central goal of the AMMOD project is to automatically collect all observed data in a central repository (the *AMMOD Portal*, https://data.ammod.de, accessed on 19 October 2022), which will eventually be accessible to biologists and the general public. For SOCRATES, we ensure this by uploading the captured raw data via the CoAP protocol [62] to the *AMMOD base station* [1,2], if available at the current location, or directly to the AMMOD Portal otherwise. The AMMOD base station takes the role of scheduling and prioritizing data transfer from different sensors according to the energy available from energy harvesting. Once the raw data are uploaded to the AMMOD Portal, a server runs the instance segmentation (see Section 3.3) and distance estimation (see Section 3.4) workflows. To increase throughput and energy efficiency, the server is equipped with an NVIDIA Quadro RTX 6000 GPU to accelerate neural network inference. Both methods are packaged as *Docker* images to simplify dependency management and updates. The resulting instance masks and distances are then again uploaded to the AMMOD Portal and are available for further analysis by biologists. This data flow is fully automated and visualized in Figure 9.

## 5. Conclusions

We propose SOCRATES as a novel camera trap approach for automated wildlife monitoring. SOCRATES employs stereo vision to improve animal detection and abundance estimation using depth information. SOCRATES comprises a flexible hardware architecture, as well as a comprehensive deep learning approach for animal detection. The depth information obtained using stereo vision improves the localization accuracy of animals in an instance segmentation setting, for example, by 3.23% in bounding box ${\mathrm{mAP}}_{75}$. The validity of these improvements was underscored by performing 10-fold cross-validation. Similar improvements on the Cityscapes instance segmentation task showed that this effect is neither limited to grayscale images, a single object category, nor fixed cameras such as SOCRATES. The animal localization provided by object detection is combined with the depth information provided by SOCRATES to measure reliable animal observation distances, which are required to estimate animal abundance using methods such as CTDS [24]. SOCRATES achieves this in a fully automated process and independently of large training datasets for monocular depth estimation based on deep learning [26]. We successfully modeled sensible detection probability in a wildlife enclosure using SOCRATES. Future work will use SOCRATES to perform automatic abundance estimation in the wild and compare the results with competing monocular approaches [25,26]. In an effort to make SOCRATES accessible to biologists and ecologists, we openly provide our raw and labeled data, code, detailed instructions, best practices, and 3D CAD models at https://github.com/timmh/socrates (accessed on 19 October 2022). We hope to pave the way for the eventual adaption of stereo camera traps by commercial manufacturers.

## Figures and Tables

**Table 1:** Comparison between SOCRATES and the widely used Reconyx HP2XC trail camera. The video length of SOCRATES is only bounded by the available persistent storage space, although we used 25 s videos for our experiments. Costs include only materials and not assembly.

	HP2XC [51]	SOCRATES
Provided Depth	✗	✓
Image Resolution	1920×1080/3 MP	1920×1080
Video Resolution	1280×720	1920×1080
Video Length	max. 90 s at 2 FPS	Up to 40 h at 30 FPS
Daytime Imaging	RGB	Near-infrared
Nighttime Imaging	Near-infrared	Near-infrared
Illumination Wavelength	940 nm	850 nm
Dimensions	14×11.5×7.5 cm	11.6×80×20 cm
Connectivity	Cellular	Cellular/W-LAN/LAN
Battery Life	Up to a year	∼9 days
Material Cost	USD 659.99	∼USD 900

**Table 2:** COCO metrics on the Plittersdorf instance segmentation dataset task using Cascade Mask R-CNN with different backbones and 10-fold cross-validation. The respective best value for each metric is highlighted in bold.

Backbone	${\mathrm{AP}}^{\mathrm{bbox}}$	${\mathrm{AP}}_{75}^{\mathrm{bbox}}$	${\mathrm{AP}}^{\mathrm{segm}}$	${\mathrm{AP}}_{50}^{\mathrm{segm}}$
Swin-L	0.5164	0.5272	0.4359	0.8328
Omnivore-L	0.5243	0.5702	0.4382	**0.8353**
+depth-awareness	**0.5399**	**0.6048**	**0.4547**	0.8147
**Backbone**	${\mathrm{AP}}_{75}^{\mathrm{segm}}$	${\mathrm{AP}}_{l}^{\mathrm{segm}}$	${\mathrm{AP}}_{m}^{\mathrm{segm}}$	${\mathrm{AP}}_{s}^{\mathrm{segm}}$
Swin-L	0.4285	0.5192	0.3856	0.1285
Omnivore-L	0.4376	0.515	0.3895	**0.1431**
+depth-awareness	**0.4699**	**0.5427**	**0.4013**	0.1138

**Table 3:** Instance segmentation results on the Cityscapes validation set. The depth-aware Omnivore-L variant clearly improves the non-depth-aware variant in all metrics. The metrics of the Swin-L backbone were obtained using the original implementation [15]. ${\mathrm{AP}}^{\mathrm{segm}}$ and ${\mathrm{AP}}_{50}^{\mathrm{segm}}$ are Cityscapes metrics; the rest are COCO metrics. The respective best value for each metric is highlighted in bold.

Backbone	${\mathrm{AP}}^{\mathrm{bbox}}$	${\mathrm{AP}}^{\mathrm{segm}}$	${\mathrm{AP}}_{50}^{\mathrm{segm}}$	${\mathrm{AP}}_{l}^{\mathrm{segm}}$	${\mathrm{AP}}_{m}^{\mathrm{segm}}$	${\mathrm{AP}}_{s}^{\mathrm{segm}}$
Swin-L		0.437	0.714			
Omnivore-L	0.415	0.439	0.700	0.716	0.394	0.214
Depth-aware Omnivore-L	**0.431**	**0.456**	**0.734**	**0.732**	**0.411**	**0.264**

## Data Availability

The data obtained using SOCRATES are freely available at https://doi.org/10.5281/zenodo.6992653 (accessed on 20 September 2022) and https://doi.org/10.5281/zenodo.7035934 (accessed on 20 September 2022).

## Abbreviations

The following abbreviations are used in this manuscript:

SOCRATES	StereO CameRA Trap for monitoring of biodivErSity
CTDS	camera trap distance sampling
RGB	red, green, blue (color channels)
IR	infrared
PIR sensor	pyroelectric infrared sensor
HEVC	High-Efficiency Video Coding

## Appendix A. Comparison with ResNet Backbones

We performed an additional experimental comparison of Omnivore-L [19] and smaller ResNet [63] backbones on the Plittersdorf instance segmentation task. In the depth-aware ResNet variants, we modified the first convolutional layer to accept four instead of three channels, but otherwise used weights pre-trained on ImageNet [17]. The implementation of the rest of the detection model and the training process remained identical (see Section 3.3). As can be seen in Table A1, the Omnivore-L backbone outperforms the smaller ResNet backbones in all metrics. Still, the ResNet models profit substantially from depth awareness. Additionally, the ResNet backbones are significantly smaller and faster, as illustrated by Table A2. This potentially makes running the ResNet-based instance segmentation model directly on SOCRATES more feasible.

**Table A1:** COCO metrics on the Plittersdorf instance segmentation dataset task using Cascade Mask R-CNN with depth-aware Omnivore-L and ResNet backbones, evaluated using 10-fold cross-validation. The respective best value for each metric is highlighted in bold.

Backbone	${\mathrm{AP}}^{\mathrm{bbox}}$	${\mathrm{AP}}_{75}^{\mathrm{bbox}}$	${\mathrm{AP}}^{\mathrm{segm}}$	${\mathrm{AP}}_{50}^{\mathrm{segm}}$
Depth-aware Omnivore-L	**0.5399**	**0.6048**	**0.4547**	**0.8147**
ResNet-18	0.3361	0.2754	0.2788	0.6669
+depth-awareness	0.3318	0.3194	0.3082	0.6408
ResNet-50	0.3744	0.3624	0.3038	0.6936
+depth-awareness	0.4122	0.3804	0.3486	0.7164
**Backbone**	${\mathrm{AP}}_{75}^{\mathrm{segm}}$	${\mathrm{AP}}_{l}^{\mathrm{segm}}$	${\mathrm{AP}}_{m}^{\mathrm{segm}}$	${\mathrm{AP}}_{s}^{\mathrm{segm}}$
Depth-aware Omnivore-L	**0.4699**	**0.5427**	**0.4013**	**0.1138**
ResNet-18	0.1585	0.3129	0.2807	0.0987
+depth-awareness	0.2433	0.3487	0.2615	0.0563
ResNet-50	0.2227	0.3572	0.3108	0.0703
+depth-awareness	0.2475	0.4128	0.3009	0.0724

**Table A2:** Benchmarks of the two ResNet [63] and the Omnivore-L [19] backbones on a single NVIDIA Quadro RTX 6000.

Backbone	Parameters	Training Duration (h)	Inference Time Per Image (ms)
ResNet-18	11M	3.5	35.0
ResNet-50	24M	5.4	102.2
Omnivore-L	196M	28.1	471.1
